# Supplementary material for: Annual HIV screening rates for HIV-negative men who have sex with men in primary care
Source: PLoS One. 2022 Jul 14;17(7):e0266747. doi: 10.1371/journal.pone.0266747 (PMC9282649; doi:10.1371/journal.pone.0266747)
Supplement: Attachment — Submitted filename: response to reviewers_MP0107_cbs edits-dmh edits.docx [file pone.0266747.s001.docx]

January 17, 2022

Elisa Panada

Associate Editor

PLoS ONE

Re: PONE-#-21-22682: Annual HIV screening rates for HIV-negative men who have sex with men in primary care https://www.editorialmanager.com/pone/default1.aspx

Dear Dr Panada,

We are happy to provide the revisions to our manuscript as requested by the reviewers.

| Reviewer # 1 | Response to reviewer | Page |
| --- | --- | --- |
| Please provide a justification for limiting the sample to ages 18 to 45. The CDC recommendations are not limited to men in these age groups, so I see no reason not to include men of all ages. In fact, I would expect disparities in rates of testing by age to be even greater if older men had been included in the sample. | We thank you for this question. We agree that older men are equally important for HIV screening and would probably be even less screened than younger men, but we restricted our age range due to limited resources for data acquisition and analyst time. | - |
| Please also provide some discussion about how reliably data on sexual orientation are actually reported in the EHR. The authors do not mention any missing data on this variable, but I can't believe that there were no cases with sexual orientation excluded. Depending on the size of the sample, it may be necessary to run the analyses with three groups (MSM, non-MSM, and missing). | Sexual orientation was determined based on the person’s response to an intake question asking them to disclose if they have a sexual partner. Data were extracted from the EHR only for patients who met the inclusion criteria of having a record of a sexual partner: male, female, none, or both. We cannot attest to the accuracy/reliability of self-disclosed sexual activity.  We acknowledge this limitation in lines 219-xxx: “**Patients with missing data for sexual partner were not included in our analysis**.” | methods |
| Please reference the tables/figures in the narrative portion of the text to help guide the reader. | The table/figure references are included in the narrative portion in bolded green font. |  |
| I do not understand why the authors chose to examine the data across years. Is there any reason to expect an increase in HIV screening over the three-year period? I see no justification for that and it does not make sense to me | The data were split into one year segments because the CDC recommendation is for eligible MSM to be screened at least once annually if his HIV status was negative or unknown. We used men with fixed encounter dates within 12 months to define the eligible population for screening in that year. We were able to extract the sexual orientation data for three distinct years. There was not a hypothesized change in screening rates over time; instead, we wanted to establish baseline annual screening rates, which we felt would be more accurate over a several-year period rather than a single year.  We presented the annual HIV screening rates for both MSM and non-MSM and found no difference year over year. This baseline is essential for future quality improvement projects to increase screening rates. We have added this in the discussion.  Lines 189-190: “**In addition, by analyzing data over three years, we find that HIV testing rate for MSM are consistently low, thus establishing a reliable baseline annual testing rate**.”  Lines 195-xxx: “**These baseline annual testing and counseling rates are important to establish for future quality improvement interventions to meet CDC or USPSTF screening goals**.” |  |
| The question is about an individual's receipt of an HIV screening test, so I would have expected the analysis to focus at the individual level to see if any screening had occurred, controlling for the number of days/years the individual was included in the sample. Those with data across all three calendar years would have more opportunity to have a screening test than those who were in the sample for only one year. | Thank you. To clarify, analysis was performed at the person-year level. Individuals were included in each year period based on whether or not they had a record of any encounter during the time frame. If an individual did not have an encounter in a given year, they were not included in the denominator for screening rates, nor did they contribute to models for that year. Screening is meant to be done annually, and so individuals were included in each year as long as they had at least one encounter, regardless of prior screening status. |  |
| The finding of significant differences by year but no significance by the CMH test was confusing at first, because the authors provided no justification for expecting changes in screening patterns by year. The approach to analysis that I have suggested seems to me more in line with the stated objectives of the study. | We have stated the purpose of CMH testing in the Methods section in lines 112-114. However, we agree with the reviewer that the interpretation of the CMH test result was not clearly stated. We have revised the results section with the following, which more appropriately points out that the test finds consistent differences between the MSM and non-MSM ordering rates across the three time periods rather than suggesting we were looking for changes in rates across time.  Lines 137-xxx: “**The CMH test for homogeneity of odds ratios across the three years was not significant (p-value=0.19), suggesting this difference in HIV test ordering rates between groups was consistent across the three time periods**.” |  |
| On a related note, in line 190, the authors note that the lack of an increasing in screening is "a disquieting finding." The low level of testing overall is certainly disquieting, but since the authors have not indicated a reason to expect an increase in annual rates of screening, this finding in and of itself is not especially "disquieting." | We thank the reviewer and share his/her level of dissatisfaction with the wording. It is correct that we did not have reason to expect any change in rates of annual screening, as our intent was to establish baseline screening annual rates prior to initiating quality improvement interventions at our clinics. We are changing the wording to reflect that the rates did not increase over 3 years, but instead were consistent.  Lines 189-190: “**In addition, by analyzing data over three years, we find that HIV testing rate for MSM are consistently low, thus establishing a reliable baseline annual testing rate**.” |  |
| Lines xxx-xxx (previously 165-166) appear to be missing something - the sentence is incomplete | We used a green font to highlight the table and figures. It is apparent that the green color was not visible in the reviewer’s copy of the manuscript. The sentence is complete: “Figure 1 illustrates interaction results through the predicted probability of HIV test being ordered under different levels of group, race, and self-reported illegal drug use.” |  |
| The Limitations section should be expanded to include more discussion of the reliability of the methods used.  Do the authors have any means to determine how regularly clinicians include in their notes discussions with patients about matters such as HIV testing, especially if the patient refused to be tested? | This is a retrospective review of medical records, and, as has been well-established, not all information is recorded in a clinic visit that a research visit would require. We include in our methods section our process for determining how clinicians include discussion with patients about HIV testing in their notes.  Lines 104-106: “**For patients who did not have an HIV test ordered, we additionally searched encounter notes for the terms “HIV” and “STI” in conjunction with “counsel”, “screen” and “test”. The presence of these terms could indicate a pertinent discussion about testing for HIV**.”  Additionally, we acknowledge the limitations of documentation reliability in the limitations section beginning on line 220 and have added additional clarification as follows.  **Lines 223-xxx: “Furthermore, while a proportion of encounter notes suggest discussion of HIV for MSM who did not have a test order, we cannot verify how accurately EHR documentation reflects actual patient-provider discussions**.” |  |
| Please provide details within the text regarding which characteristics the study's sample "matches the distribution in the state of Michigan." Is that only in regards to the percentage of men identified as MSM (4.1 %) or is that across other demographic characteristics? If it is only in terms of the percentage, then the authors' claims of generalizability cannot be substantiated. It could be that the percentage is similar but that the study sample includes a higher percentage of white MSM than found in the state as a whole. | Our results are not weighted by population proportions which would have been the more accurate way to describe generalizability of our results to others in Michigan. Therefore, we have moved this paragraph to a limitation agreeing that we cannot state these are generalizable to a wider population other than our clinic panels.  Lines 224-225: “**Methodologically, while a strength of our work is that our population distribution of MSM and non-MSM matches the population distribution in the state of Michigan (MDHHS 2021), our results are not weighted by population proportions across other demographic characteristics. Therefore, we cannot generalize results to a wider population than our clinic panels.**” |  |
| **Reviewer # 2** |  |  |
| on page 6, line 81, you mention non-MSM and state bi-sexual men. I understand those who may not be aware of their sexual identity, but was curious why you did not include bi-sexual men as MSM? Are you suggesting here that the bisexual men may not have engaged in a sexual encounter with another man or are you just directly placing bisexual men in a "non MSM" category? | To clarify, individuals were identified as MSM in our study if they had ever indicated having a male partner, regardless of whether or not they ever had a female partner. So, in that respect we did include bi-sexual men in our MSM group. We have clarified this in the methods section.  Lines 98-xxx: “**Eligible patients were grouped based on reported sexual partners. Individuals who reported having a male sexual partner or both a male and female partner were included in the MSM group. The non-MSM group included individuals who reported only a female partner and those with neither male nor female partners**.” |  |
| Another question I had was the distribution of race/age based on practice setting. This is clearly not a focus of your work, but as you noted, STI clinics and ER's are frequently used sites; however, because you are the first to describe this data, did you see any associations with race/ethnicity and practice setting? Again, a question I had while reading that may have implications for clinical intervention implementation. And as a total side note, it would be interesting to see if the age/race/ethnicity/gender of the provider showed any significant outcomes | We did collect data on the practice setting and on the age/race/ethnicity/gender of both the providers and patients. We agree that this would be interesting for future analysis but is beyond the scope of this initial study. |  |
| it took me a few seconds to understand table 1, which made me wonder if it would make for a more clear presentation to have the M and SD in the column header. That is the format I think most readers are accustomed to seeing. | Yes, we understand that if all of the data were means and standard deviations, then the labeling would be most appropriate in the header. However, the bottom half of the table presents data in number and percent, therefore, we must keep the labeling as it is. |  |
| Finally, please consider changing your conclusions section to a Discussion, followed by your important conclusion statement. You have many important points to address and separating the discussion from your suggested conclusions may better frame the entirety of your discussion as well as making the clinical implications stand out to readers. | Thank you. We agree with this reviewer’s suggestion and have changed our conclusions section to discussion and added our conclusion statement beginning on line 231. |  |
| Thank you again for allowing me to review this manuscript. I enjoyed reading it and can clearly see the importance of this study. | Thank you for your kind words. |  |
|  |  |  |
