# Supplementary material for: Annual HIV screening rates for HIV-negative men who have sex with men in primary care
Source: PLoS One. 2022 Jul 14;17(7):e0266747. doi: 10.1371/journal.pone.0266747 (PMC9282649; doi:10.1371/journal.pone.0266747)
Supplement: Attachment — Submitted filename: response to reviewers.docx [file pone.0266747.s002.docx]

March 10, 2022

Dawn K Smith

Academic Editor, PLOS ONE

Re: PONE-#-21-22682R1: Annual HIV screening rates for HIV-negative men who have sex with men in primary care

Dear Editor Smith,

Thank you for the opportunity to respond a second time to Reviewer #1. We apologize for any miscommunication between our responses and the understanding of our comments.

| Reviewer Comment | Response | Page |
| --- | --- | --- |
| The authors have clarified the source of information regarding sexual orientation. However, there remains some confusion. Over what timeframe was the question regarding sexual partner asked? Over the past year? Lifetime? | The question that is asked of each person is “What is the sex of your partner?” and the boxes to check are one for female, one for male, and a comment box. This sexuality question is meant to be reviewed at every visit but is often answered only at the first visit. A person can check none, one, or both boxes each time the question is updated.  We have changed the sentence to say:  **Eligible patients were grouped based on reported sexual partners at the most recent disclosure**. | 5 |
| The way I have seen this question asked in a clinical setting is in regard to sexual preference (male, female, or both). Maybe preference rather than "having a sexual partner" question refers to whether or not the patient has a current sexual partner)? In any case, a current sexual partner is not necessarily indicative of sexual orientation, and that should be mentioned in the Limitations section. | We do not intend for the response to the question of who one has sex with to indicate sexual orientation. We define MSM literally to be men who have sex with men, not an orientation. |  |
| In the Materials and Methods section, the authors state that the non-MSM group includes "those with neither male nor female partner." I’m assuming that the authors mean that the person indicated that they were not sexually active but the wording is awkward since it could be taken to mean that the patient-reported sexual activity with a non-human partner. | Thank you for drawing this inappropriate attribution to our wording to our attention.  We have changed the sentence to read:  **The non-MSM group included individuals who reported only a female partner or no partner.** | 5 |
| The discussion is also confusing because, in their response to reviewer comments and Discussion, the authors now state that "Patients with missing data for a sexual partner were not included in our analysis." If the authors mean that a person is missing data on sexual activity, then it would be better to simply say that in the methods section. | We have added this sentence to the methods section:  **Patients who did not answer the question were excluded from the study.** | 5 |
| The authors should state the number of cases excluded due to missing data. that is what the authors intended to say | We apologize that this is still a confusing point in the manuscript and have attempted to clarify the text to make it more clear. We extracted data from the EHR on all eligible men who indicated being sexually active**. If they were not sexually active, they would not have completed partner information and therefore not be included in the extraction**. We included all men who were extracted and grouped them into either those who had some indication of a male partner vs. those who had only female or no known partner, but sexually active with humans. |  |
| How was the search of the encounter notes performed? Given the large number of encounters included in the study,  I'm assuming this process was automated. The authors should provide more details regarding the methods of the search. | We appreciate the reviewer’s concern for accuracy. We state in the methods that the terms HIV counsel…, HIV screen… HIV test… as well as STI counsel… STI screen… and STI test were programmed in the automated search. We feel that this is clear and that providing more coding detail would be confusing to the reader with no additional value. | 6 |
| The description is also unclear because the authors specifically reference "rates of HIV discussion among MSM."  Table 2 suggests that the search was performed for all patients included in the study (i.e., including for non-MSM), but the description suggests otherwise. If the same procedure was followed for both groups (which I'm sure it was), then that should be stated clearly. | As noted below, we have clarified that the same procedure was used for MSM and non-MSM by stating:  **The same procedure was completed separately for non-MSM.** | 6 |
| Similarly, when describing the calculation of the annual testing rates, the authors only mention the calculation for MSM. For the sake of completeness, please indicate that the same procedure was completed separately for non-MSM. | Thank you. We have added this sentence:  **The same procedure was completed separately for non-MSM.** | 6 |
| Table 1 as reformatted remains confusing as the table headers do not align with the columns and the p-value column appears to be missing. I disagree with the authors' statement to the second reviewer that "we must keep the labeling as it is." There are ways to improve the presentation of the data and that should be done. Tables and Figures should stand on their own, so all necessary clarifications should be made | We profusely apologize for Table 1. We agree that the table as presented to you was massively mis-formatted. We have corrected this issue. | 7 |
| In the first paragraph of the Results section, please take out the statement, "... consistent with state-specific data for generalizability." Per my earlier comments and the authors' acknowledgment, this statement is confusing and not valid in any case. Please also remove the reference to generalizability from the discussion section (i.e., the paragraph that starts,  "Methodologically, while a strength...") or at least limit it the context of the clinical sites | We apologize. We have now removed this statement and its references from the results section and from the discussion section. | 6 |
| In the Discussion section, the authors state, "Our results also indicate that multi-level interventions with the health system, primary care physicians, and men themselves could be necessary to increase the HIV test ordering rate."  The study does not address the specific barriers to testing, so this statement (even if true) is not specifically substantiated by the study. The authors may justify this statement on the basis of other research, but not on the basis of the study as reported. | We thank you for noting this obscurity. We have changed the first two sentences of this paragraph to :  **Our results suggest that current systems are inadequate. We hypothesize that multi-level interventions with the health system, primary care physicians, and men themselves could be necessary to increase the HIV test ordering rate.** | 10 |
| Please provide the tables describing the logistic regression models. The discussion is difficult to follow without data to  reference. | We have provided Table 3 which provides the details of the logistic regression model. |  |

We are grateful for your attention to detail and sincerely feel we have made all the changes and clarifications asked.

Sincerely,

Diane M Harper MD MPH MS

Professor

University of Michigan
